# The Effect of Intravenous Dexmedetomidine During Surgery in the Prevention of Shivering After General Anesthesia in Patients Undergoing Spinal Surgery: A Randomized Clinical Trial

**DOI:** 10.5812/aapm-159077

**Published:** 2025-05-18

**Authors:** Mehdi Shokri, Zhale Bakhtiari, Bita Kargar, Amirhossein Hajialigol

**Affiliations:** 1 School of Medicine, Tabriz University of Medical Sciences, Tabriz, Iran; 2 Tabriz University of Medical Sciences, Tabriz, Iran; 3 Islamic Azad University of Medical Sciences, Tehran, Iran; 4 Alborz Office of Universal Scientific Education and Research Network (USERN), Alborz University of Medical Sciences, Karaj, Iran

**Keywords:** Spinal Surgery, General Anesthesia, Prone Position, Shivering, Dexmedetomidine

## Abstract

**Background:**

Postoperative shivering is an involuntary, spontaneous, and repetitive contraction of skeletal muscles that causes patient restlessness, increased oxygen consumption, wound infection, surgical bleeding, and cardiac events. Patients undergoing spine surgery in the prone position are particularly susceptible to hypothermia.

**Objectives:**

Given the importance of controlling postoperative shivering in these patients, the present study aimed to investigate the effect of intraoperative dexmedetomidine (Dex) infusion in preventing shivering after general anesthesia in patients undergoing spine surgery in the prone position.

**Methods:**

In this double-blind randomized clinical trial, 60 American Society of Anesthesiologists (ASA) class I or II patients undergoing vertebral surgery in the prone position were enrolled. Patients in the study group (n = 30) received Dex infusion during surgery, while those in the placebo group (n = 30) received an equivalent volume of 0.9% normal saline. Hemodynamic variables, frequency and severity of shivering, and drug side effects were recorded.

**Results:**

The mean arterial pressure (MAP) at 90 minutes (P = 0.022), immediately before extubation (P = 0.001), and after extubation (P = 0.001), as well as HR values at 60 minutes (P = 0.020), 90 minutes (P = 0.001), immediately before extubation (P = 0.001), and after extubation (P = 0.001), were significantly lower in the study group compared to the placebo group. The frequency of bradycardia (26.7% vs. 0%, P = 0.002) and hypotension (20% vs. 0%, P = 0.012) was significantly higher in the study group. At all evaluated times, the mean body temperature in the study group was significantly higher than in the placebo group (P < 0.05). The frequency (10% vs. 30%, P = 0.003) and intensity (P = 0.001) of shivering in the study group were significantly lower than in the placebo group.

**Conclusions:**

This study demonstrated that the preventive use of Dex infusion during surgery reduces the frequency and severity of postoperative shivering in patients undergoing spinal surgery in the prone position. However, this method was associated with hypotension and bradycardia in some patients.

## 1. Background

Post-surgery shivering (PSS) is observed in over 60% of individuals following general anesthesia (1). This phenomenon serves as a critical thermoregulatory defense mechanism in adults and is characterized by spontaneous, asynchronous, and involuntary contractions of skeletal muscles, known as fasciculations (2). The onset of shivering can occur anywhere from 5 to 37 minutes after the cessation of anesthesia (3). It is recognized as an unpleasant and frequently encountered complication post-surgery, manifesting in varying intensities, from mild skin goosebumps to severe, continuous muscle contractions. Such tremors can adversely affect the quality of recovery following anesthesia (4). Additionally, shivering may disrupt the monitoring of electrocardiograms (ECGs), blood pressure (BP), and arterial oxygen saturation. It also leads to increased oxygen consumption, lactic acidosis, and carbon dioxide production, ultimately diminishing patient satisfaction and heightening discomfort (5). Shivering is associated with heightened intraocular and intracranial pressures, as well as an increased mortality rate among patients with cardiovascular ailments. This occurs because shivering boosts cardiac output, resulting in a surge in metabolic heat production — up to 600% above baseline levels (3, 4). Patients often express that the discomfort from shivering is significant, with some even claiming that the cold feeling it generates is more intolerable than the pain of surgery. Moreover, shivering may aggravate postoperative pain by stretching surgical incisions (6).

Hypothermia during anesthesia follows a specific pattern: A rapid decrease in central body temperature, followed by a slow linear decline, and finally stabilization of central body temperature (7, 8). The mechanisms of shivering in patients undergoing surgery include heat loss during surgery, increased sympathetic tone, pain, and systemic release of pyrogens (8, 9). Non-thermoregulatory shivering occurs in normothermic patients in response to anesthetics and postoperative pain (1). The prone position is commonly used to access the head, neck, and posterior spine during spine surgery (10). Patients undergoing spine surgery in the prone position are prone to hypothermia due to the long duration of the surgery and the large surface area of the surgical field. A prevalence of shivering between 50% and 80% after spine surgery has been reported (11). Intraoperative hypothermia is associated with ocular complications during spine surgery in the prone position (11, 12). Given the effects of hypothermia and postoperative shivering, preventing its occurrence and ensuring timely treatment are among the primary goals in anesthesia (13). Despite its clinical importance, the mechanism of shivering remains poorly understood, and a gold standard for its treatment and prevention has yet to be defined (1, 14).

Mechanical methods for the prevention and treatment of hypothermia include increasing the temperature of the operating room beyond its standard level (approximately 84°F), heating and humidifying breathing gases, warming intravenous (IV) fluids (including blood) to match the patient’s body temperature, skin warming techniques, and using gastric lavage and peritoneal washing (6, 13, 15). However, non-pharmacological interventions offer inadequate control of central hypothermia, necessitating pharmacological approaches to treat and prevent shivering (16).

Several classes of pharmacological agents — including opioids, 5-hydroxytryptamine 3 receptor antagonist (5-HT3) receptor antagonists (e.g., ondansetron), α2 receptor agonists, and possibly N-methyl-D-aspartate receptor antagonists (e.g., ketamine) — modulate central thermoregulatory mechanisms. Most of these agents also possess analgesic and sedative properties (1, 17). Currently, the most commonly used drug to treat shivering is meperidine, a synthetic narcotic (18). Given the adverse effects of opioids, such as respiratory depression, pruritus, nausea, vomiting, and complications associated with pethidine (e.g., tachycardia), the need for effective alternative medications is evident (19).

Although dexmedetomidine (Dex) is among the agents used for treating shivering (20), its intraoperative infusion effects for preventing shivering after spinal surgery have not been thoroughly evaluated.

## 2. Objectives

The present study aimed to investigate the effect of intraoperative Dex infusion on the prevention of shivering following general anesthesia in patients undergoing spine surgery in the prone position.

## 3. Methods

The present study was conducted over a four-month period in 2024 at Imam Sajjad Educational and Treatment Center in Tabriz. The study protocol was approved by the Medical Ethics Committee of Azad University of Medical Sciences, Tabriz Branch, and written informed consent was obtained from all patients. A total of 60 patients, aged 20 - 60 years, with American Society of Anesthesiologists (ASA) physical status class I or II, undergoing spinal surgery in the prone position, were included in the study.

All patients fasted for 8 hours prior to the start of anesthesia. In the operating room, after securing IV access using an 18-G catheter and initiating IV fluid infusion, standard monitoring was established, including ECG, non-invasive blood pressure (NIBP), and pulse oximetry. Baseline values for systolic blood pressure (SBP), diastolic blood pressure (DBP), mean arterial pressure (MAP), heart rate (HR), and peripheral oxygen saturation (SpO_2_) were recorded.

Following pre-oxygenation and premedication with midazolam 0.03 mg/kg and fentanyl 2 μg/kg, general anesthesia was induced using propofol 2 mg/kg and atracurium 0.6 mg/kg. A spiral cuffed endotracheal tube of appropriate size was inserted. The patients were then placed in the prone position, and anesthesia was maintained with isoflurane at a monitoring care anesthesia (MAC) of 0.8 - 1.5% and 50% N2O in oxygen. Mechanical ventilation was used to maintain end-tidal carbon dioxide (ETCO_2_) at 35 ± 5 mmHg. Surgery was initiated once the Bispectral Index (BIS) reached a range of 40 - 60.

Patients were covered with surgical drapes during anesthesia but were not actively warmed. The operating room temperature was maintained between 20 and 23°C. During surgery, additional doses of fentanyl and atracurium were administered as needed for analgesia and muscle relaxation.

Patients who met all of the following conditions were included: (1) Age between 20 and 60 years; (2) American Society of Anesthesiologists physical status I or II; (3) undergoing spinal surgery in the prone position. Patients were excluded if they met any of the following conditions: (1) Hyperthyroidism; (2) cardiac or respiratory disease; (3) mental disorder; (4) initial body temperature greater than 37.5°C or less than 36.5°C.

Patients were randomly assigned to two study groups. Randomization was performed using a computer-generated method and sealed numbered envelopes by an individual blinded to the study. The patient, the surgeon, and the data-collecting student were unaware of the group assignments.

Patients in the study group (Dex group, 30 cases) received a bolus dose of Dex at 1 mcg/kg in 10 mL of normal saline, followed by a continuous infusion at 0.5 mcg/kg/h. Patients in the placebo group (30 cases) received an equivalent volume of 0.9% normal saline for both the bolus and infusion. Study solutions were prepared in coded syringes by an anesthesiologist not involved in intraoperative care or postoperative management. The infusion was stopped at the beginning of subcutaneous closure. All patients received 1 gram of paracetamol (perfalgan, 100 mL, Bristol Myers Squibb) in 100 mL of normal saline over 15 minutes.

Hemodynamic and respiratory parameters (MAP, HR, and SpO_2_) were recorded at the following time points: Baseline (before induction of general anesthesia), after induction, after tracheal intubation, and at 15, 30, 60, and 90 minutes intraoperatively, as well as immediately before and after extubation. Axillary temperature was recorded at the same time points.

During surgery, if bradycardia (HR < 50 bpm) or hypotension (SBP decrease > 20% from baseline) occurred, atropine and ephedrine were administered, respectively. At the end of the procedure, residual neuromuscular blockade was reversed, and patients were extubated and transferred to the post anesthesia care unit (PACU). The duration of surgery and anesthesia was recorded.

In the PACU, all patients received oxygen via face mask and were covered with a blanket. Axillary temperature was measured again at 1, 15, 30, 45, and 60 minutes postoperatively. The incidence and severity of shivering were evaluated using the Sagir et al. Scale (21), a 5-point scoring system: (0) No shivering; (1) piloerection or peripheral vasoconstriction without visible shivering; (2) muscle activity in one muscle group only; (3) muscle activity in more than one muscle group, not generalized; (4) generalized shivering involving the entire body. Patients who exhibited shivering of grade > 2 received IV meperidine at 0.5 mg/kg.

Finally, perioperative drug- and anesthesia-related complications [hypoxia (SpO_2_ < 90%), bradycardia, tachycardia, hypotension, hypertension, nausea and vomiting, and drowsiness] were recorded and treated as necessary.

### 3.1. Determining the Sample Size

In this study, the frequency of shivering after general anesthesia in patients undergoing spinal surgery in the prone position was considered the primary outcome, and the mean body temperature in the PACU was considered the secondary outcome. The sample size was determined by assuming an approximate 20% difference in the frequency of shivering between the two study groups and using the results of the Lamontagne study (18). Considering a study power of 95% and a significance level of 0.05, and using the two-sided binomial test, a sample size of 30 patients per group (total 60 cases) was calculated.

### 3.2. Ethical Considerations

Dexmedetomidine is a commonly used drug in anesthesia and intensive care units. This study, registered under ethical number (IR.IAU.TABRIZ.REC.1403.218), was conducted after receiving approval from the Ethics Committee of Islamic Azad University of Medical Sciences, Tabriz Branch. Written informed consent was obtained from all patients, and their information was kept confidential. It was explained to participants that they were free to withdraw from the study at any time without providing a reason, should they choose not to continue.

### 3.3. Statistical Analysis

Statistical analysis was performed using SPSS 18.0 (SPSS Inc., Chicago, IL). The frequency of shivering after anesthesia served as the primary outcome for statistical analysis. Quantitative parameters such as average age, weight, height, duration of surgery and anesthesia, tympanic temperature, MAP, HR, and SpO_2_ were analyzed using the Student’s *t*-test. Categorical data including ASA physical status, use of fentanyl, ephedrine, and atropine, shivering and its severity, and perioperative side effects including drug-related complications, were analyzed using the chi-square test and Mann-Whitney U test. Intra-group comparisons were performed using repeated-measures analysis of variance. A P-value of less than 0.05 was considered statistically significant.

## 4. Results

In this randomized, double-blind clinical trial, 60 patients aged 20 - 60 years, with physical status of class I or II according to the ASA classification and undergoing spinal surgery in the prone position, were examined. Patients were randomly divided into two groups: Dexmedetomidine and placebo.

1. Age: The average age of all patients was 48.8 ± 12.3 years, with a median of 47 years. The minimum age was 24 years, and the maximum was 60 years.

2. Gender: Among the 60 patients, 29 (48.3%) were male and 31 (51.7%) were female.

3. Weight: The average weight was 76.8 ± 9.7 kg, with a median of 76 kg. The lowest weight recorded was 55 kg, and the highest was 98 kg.

4. Height: The average height was 171 ± 0.9 cm, with a median of 169 cm. Heights ranged from 154 cm to 190 cm.

5. Body Mass Index (BMI): The mean ± SD BMI of patients in the case group was 26.9 ± 3.6, and in the control group, it was 26.4 ± 3.2.

6. American Society of Anesthesiologists classification: Among the 60 patients, 32 (53.3%) were classified as ASA class I, and 28 (46.7%) as ASA class II.

7. Duration of surgery: The average duration of surgery was 113.2 ± 33.8 minutes, with a median of 100 minutes. The minimum and maximum durations were 70 and 210 minutes, respectively.

8. Duration of anesthesia: The average duration of anesthesia was 143.6 ± 33.9 minutes, with a median of 130 minutes. The shortest anesthesia duration was 100 minutes, and the longest was 240 minutes.

The demographic findings of the patients in the two study groups are shown in Table 1. The analysis of demographic variables between the two groups did not reveal any statistically significant differences (P < 0.05).

**Table 1.** Demographic Findings and Duration of Surgery and Anesthesia of Patients in Two Study Groups ^a^

Group Variant	Dex (30 Cases)	Placebo (30 Cases)	P-Value
**Age (y)**	49.4 ± 11.9	48.2 ± 12.9	0.710
**Gender**			0.796
Male	14 (46.7)	15 (50)	
Female	16 (53.3)	15 (50)	
**Weight (kg)**	75.8 ± 9.8	77.9 ± 9.6	0.421
**Height (cm)**	170.8 ± 9.5	171.2 ± 10.4	0.877
**BMI **	26.4 ± 3.2	26.9 ± 3.6	0.489
**ASA class**			0.602
I	16 (53.3)	16 (53.3)	
II	14 (46.7)	14 (46.7)	
**Duration of surgery (min)**	108.8 ± 31.1	117.7 ± 36.4	0.316
**Duration of anesthesia (min)**	139.5 ± 31.3	147.7 ± 36.4	0.355

Abbreviations: Dex, dexmedetomidine; BMI, Body Mass Index; ASA, American Society Association.

^a^ Values are expressed as No. (%) or mean ± SD.

### 4.1. Hemodynamic and Respiratory Findings

Hemodynamic and respiratory findings of patients [MAP, HR, respiratory rate (RR), and SPO_2_] were recorded at the following time points: Before (baseline) (T0), after induction of general anesthesia (T1), after tracheal intubation (T2), and at 15 (T3), 30 (T4), 60 (T5), and 90 (T6) minutes, immediately before extubation (T7), and after extubation (T8).

#### 4.1.1. Mean Arterial Pressure

**Figure 1.**
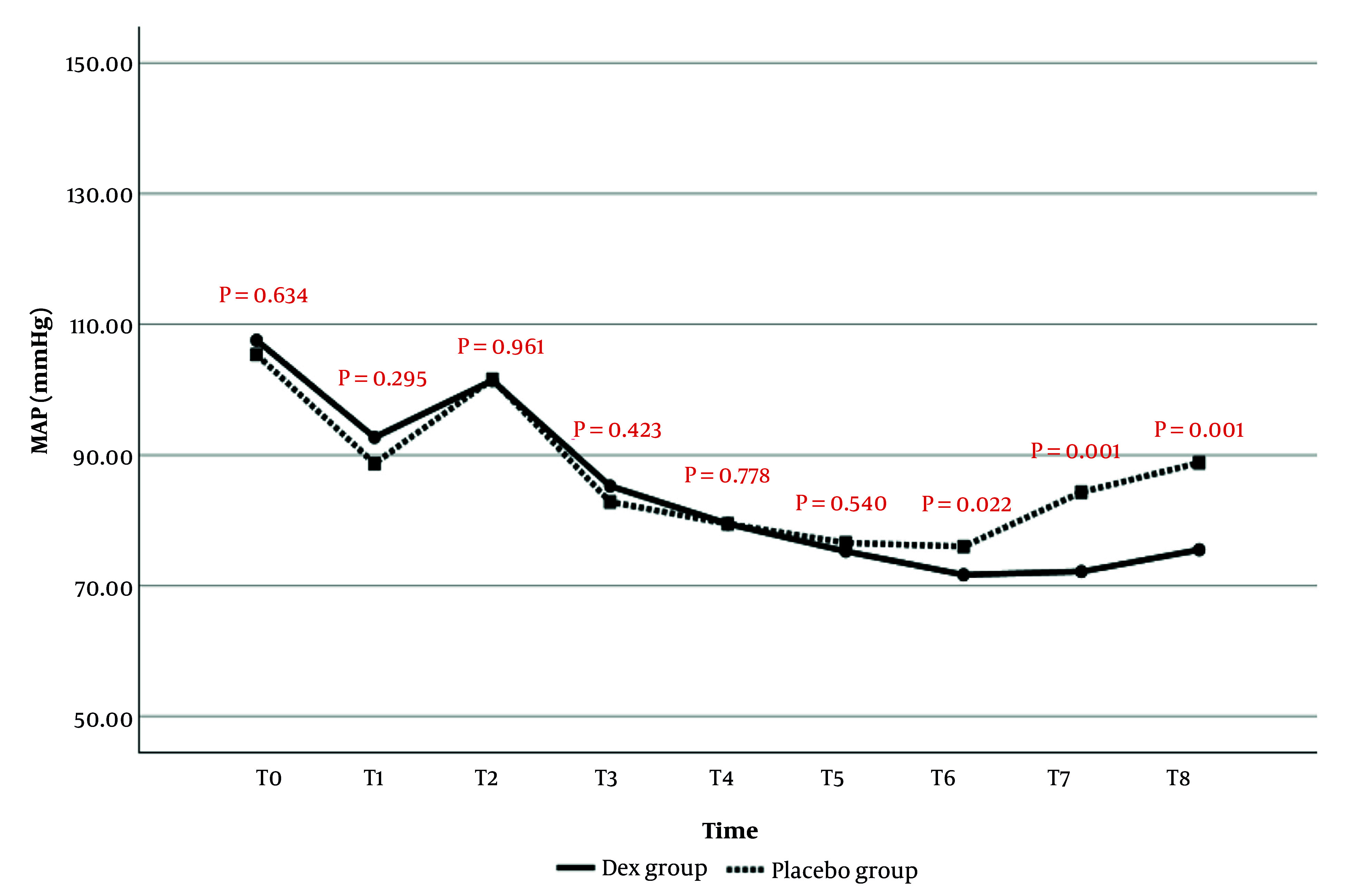
Comparison of mean arterial pressure (MAP) changes in the evaluated times in patients of two study groups

The comparison of MAP changes over time between the two study groups is presented in Figure 1. The results showed that MAP values at T6 (P = 0.022), T7 (P = 0.001), and T8 (P = 0.001) were significantly lower in the Dex group than in the placebo group. There was no statistically significant difference in MAP values at other time points between the two groups.

#### 4.1.2. Heart Rate

**Figure 2.**
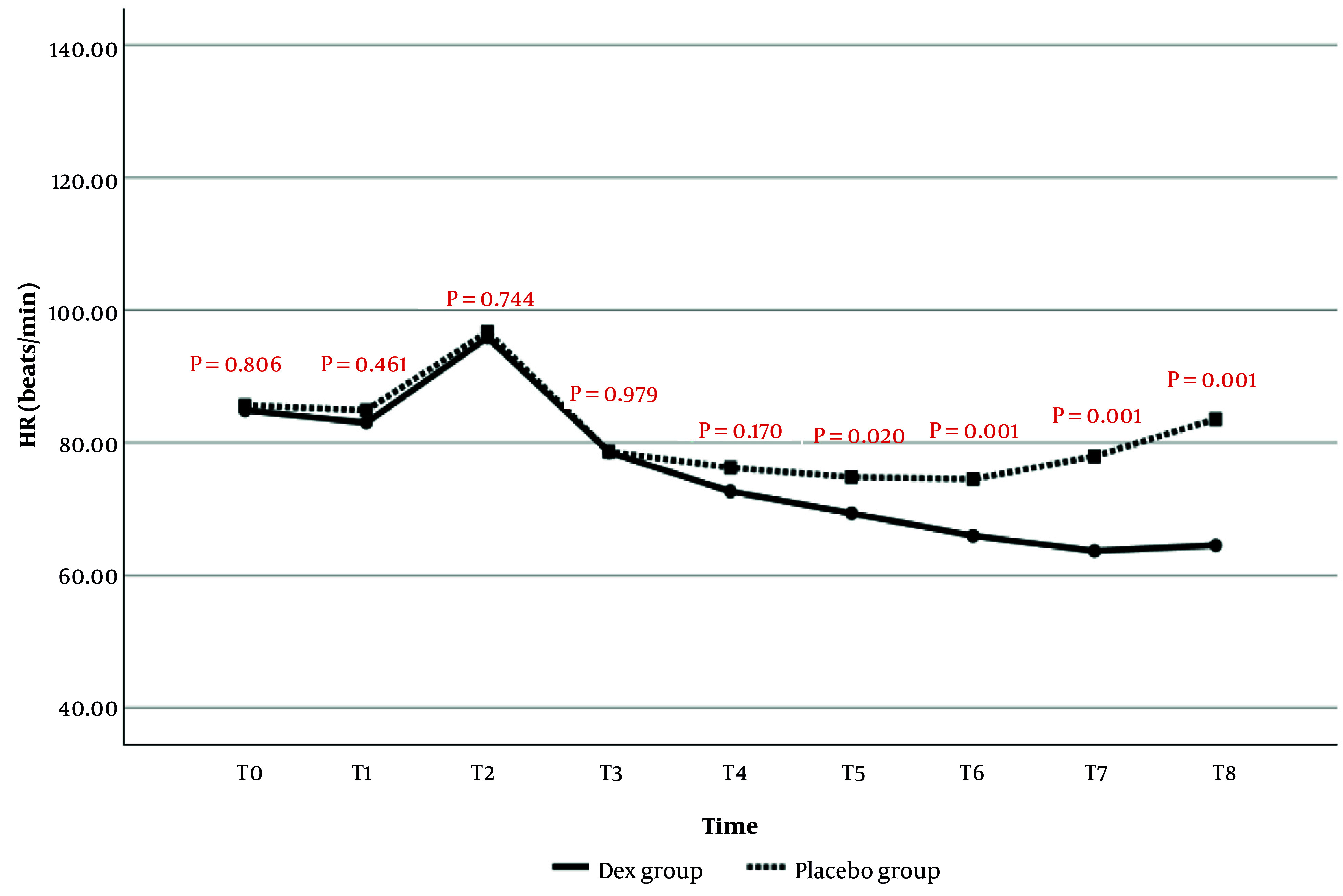
Comparison of heart rate (HR) changes in the evaluated times in patients of two study groups

The comparison of HR changes over time between the two study groups is presented in Figure 2. The results indicated that HR values at T5 (P = 0.020), T6 (P = 0.001), T7 (P = 0.001), and T8 (P = 0.001) were significantly lower in the Dex group than in the placebo group. No statistically significant differences in HR were observed at the other time points.

#### 4.1.3. Respiratory Rate

**Figure 3.**
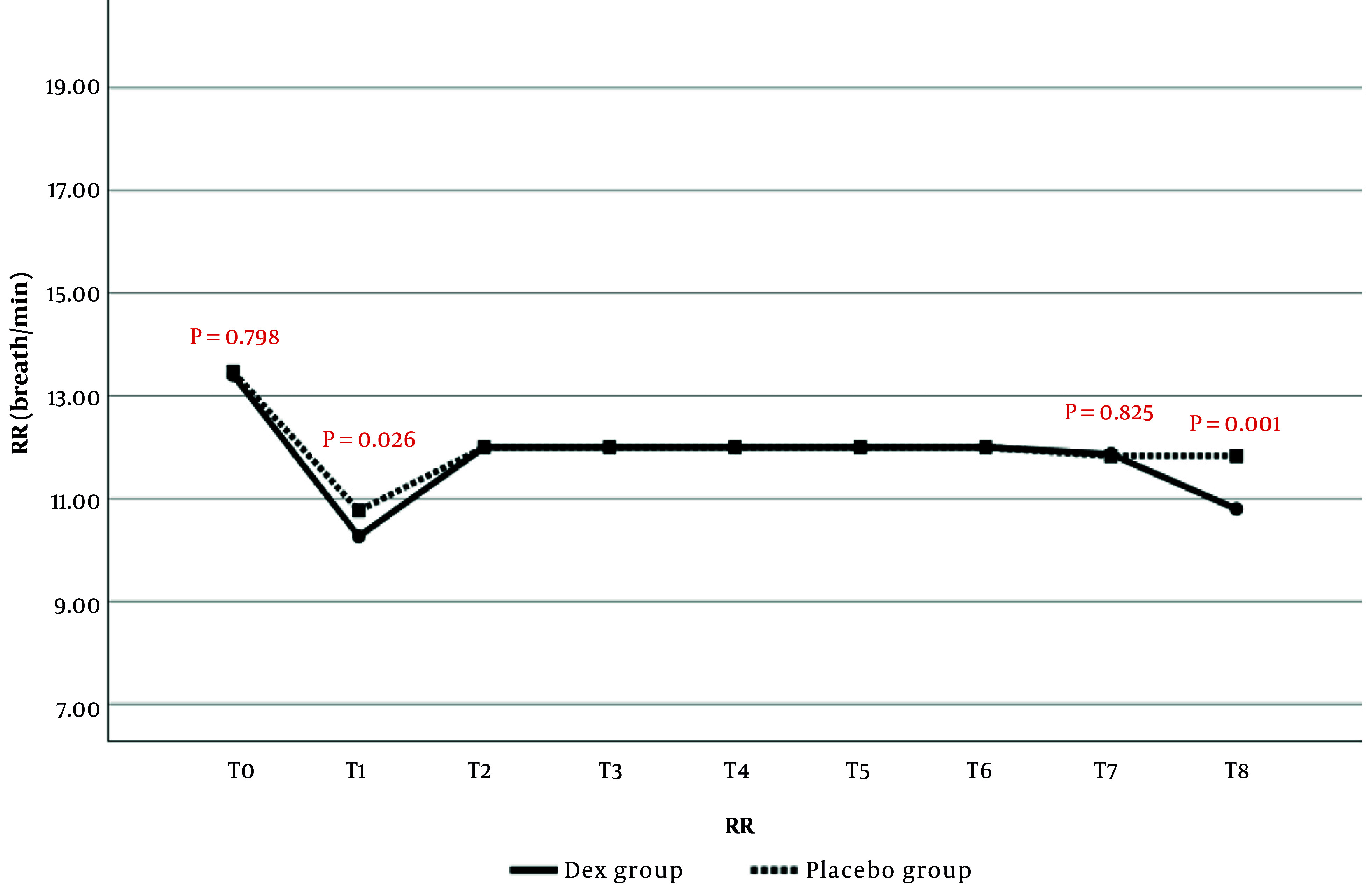
Comparison of respiratory rate (RR) changes in the evaluated times in patients of two study groups

Respiratory rate was assessed in anesthetized patients by nurses counting breaths per minute and documenting them at specified intervals. At each predefined time point (T1 to T7), RR was recorded directly from the ventilator screen. The comparison of RR changes between the two study groups is presented in Figure 3. The results showed that RR at T1 (P = 0.026) and T8 (P = 0.001) was significantly lower in the Dex group than in the placebo group. No statistically significant differences in RR were observed at the other time points.

#### 4.1.4. Arterial Blood Oxygen Saturation

**Figure 4.**
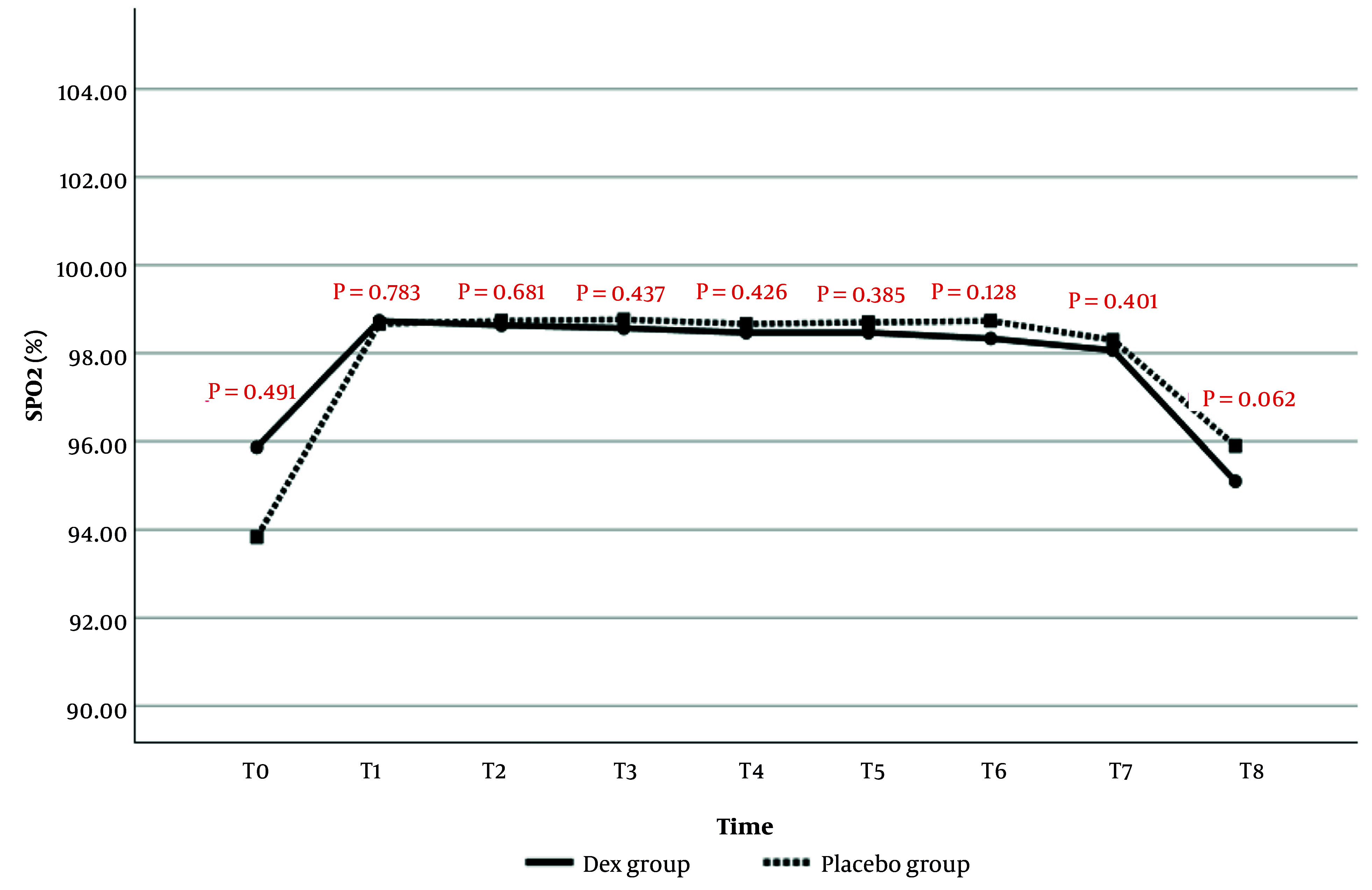
Comparison of peripheral oxygen saturation (SPO_2_) changes during evaluation times in patients of two study groups

The comparison of SPO_2_ changes between the two study groups is presented in Figure 4. The results showed no statistically significant difference in SPO_2_ values between the two groups at any time point.

#### 4.1.5. Changes in Body Temperature (Auxiliary)

##### 4.1.5.1. Comparison of Body Temperature During Anesthesia

**Figure 5.**
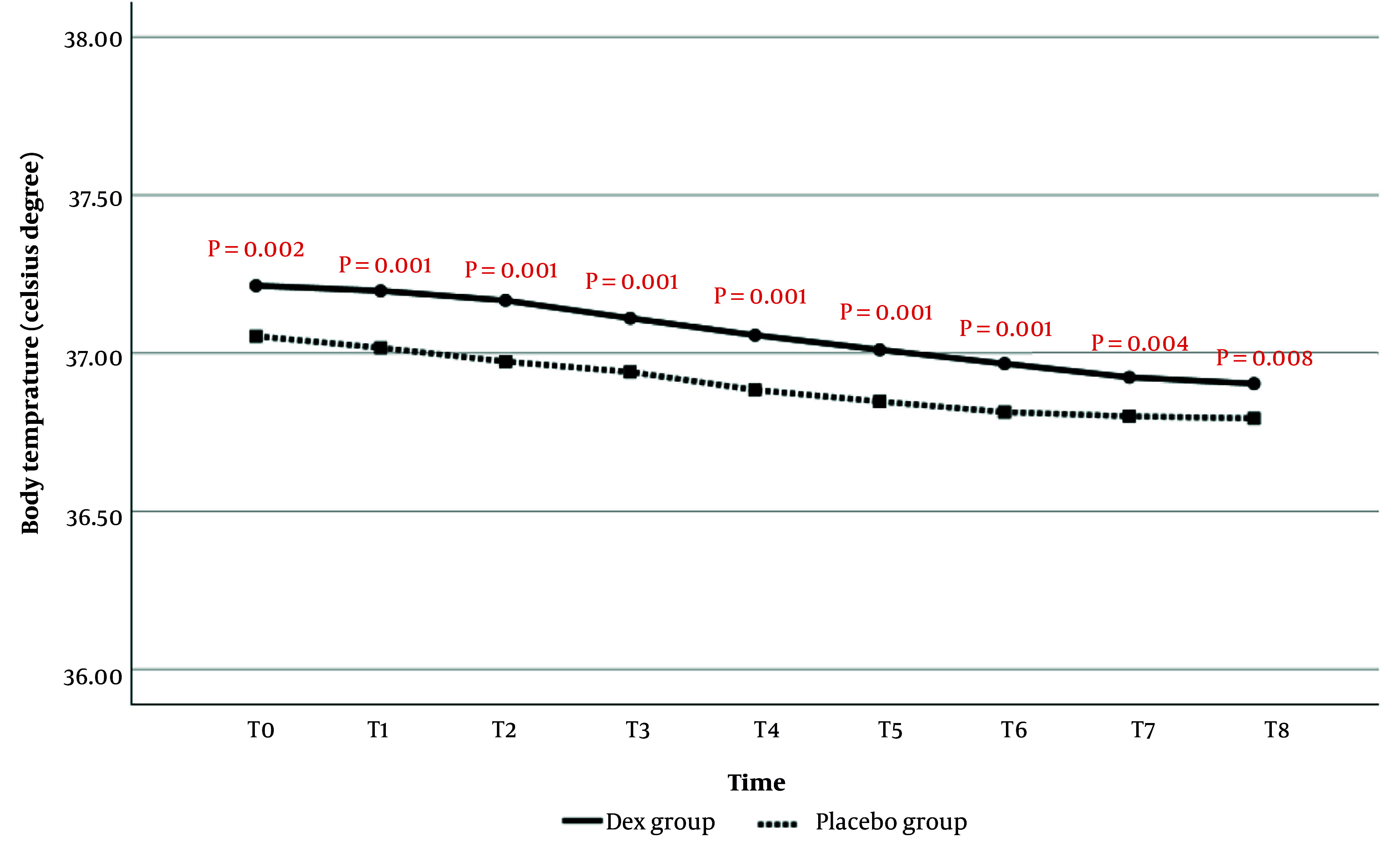
Comparison of body temperature during anesthesia at the evaluated times in patients of two study groups

Body temperature comparisons at T0 to T8 are shown in Figure 5. The results demonstrated that the average body temperature at all evaluated times was significantly higher in the Dex group than in the placebo group. Additionally, time analysis did not show a statistically significant effect of time on the reduction of body temperature (P = 0.219).

##### 4.1.5.2. Comparison of Body Temperature in Post Anesthesia Care Unit

**Figure 6.**
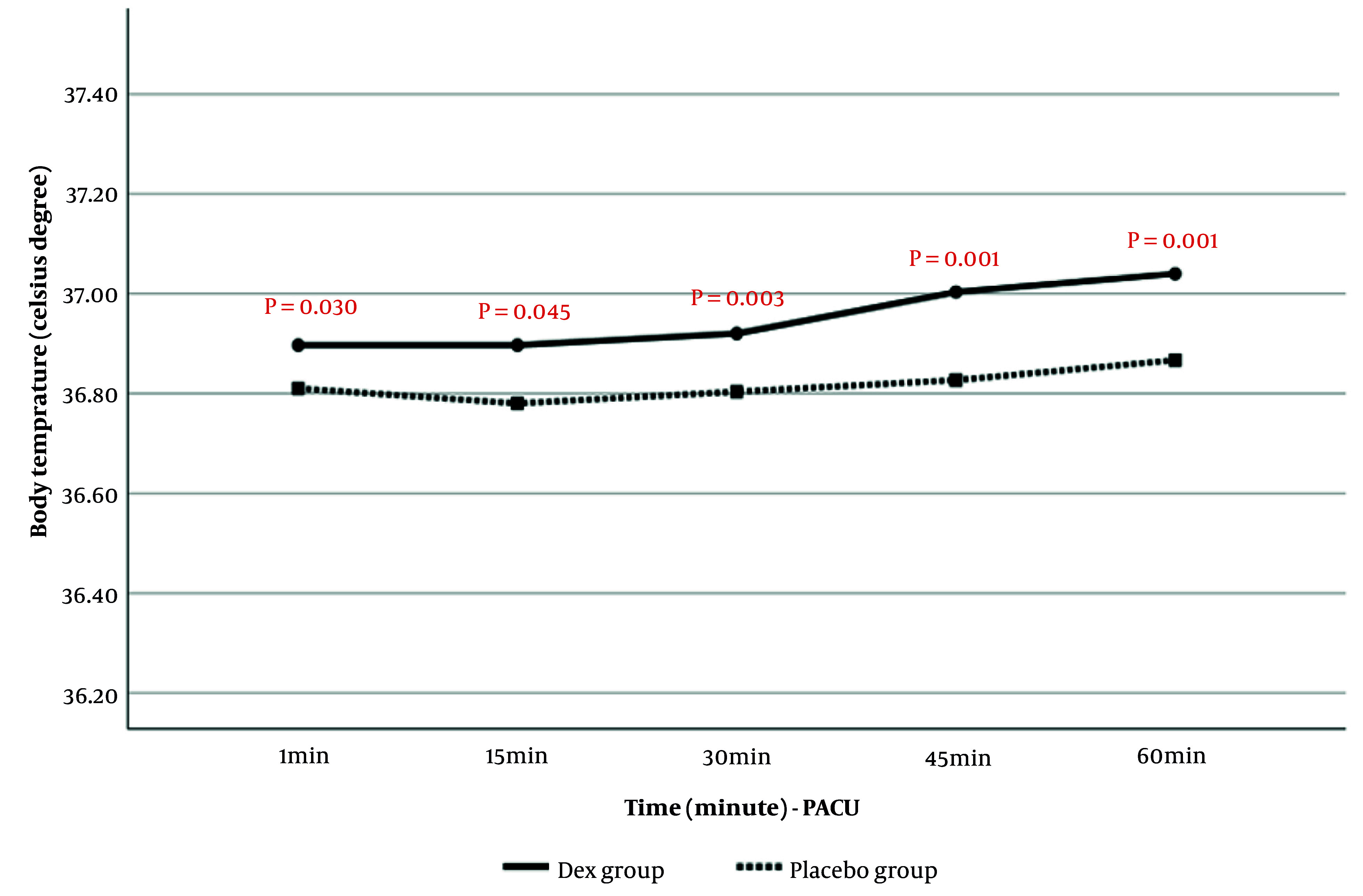
Comparison of body temperature in post anesthesia care unit (PACU) at the evaluated times in patients of two study groups

Post-anesthesia body temperature in PACU at 1, 15, 30, 45, and 60 minutes is presented in Figure 6. The results showed that body temperature in PACU was significantly higher in the Dex group than in the placebo group. Time analysis also indicated that the increase in body temperature during the assessment period was significantly greater in the Dex group (P = 0.003).

#### 4.1.6. Frequency and Intensity of Shivering

Comparison of the frequency and intensity of shivering in Dex and placebo groups is shown in Table 2. The results demonstrated that both the incidence and intensity of shivering were significantly lower in the Dex group compared to the placebo group.

**Table 2.** Comparison of Frequency and Severity of Shivering in Patients of Two Study Groups ^a^

Group Variables and Classification	Dex (30 Cases)	Placebo (30 Cases)	Total	P-Value
**Frequency of shivering**				0.003
Yes	3 (10)	9 (30)	12 (20)	
No	27 (90)	21 (70)	48 (80)	
**Shivering intensity**				0.001
I	3 (100)	3 (33.3)	6 (50)	
II	0	4 (44.4)	4 (33.3)	
III	0	2 (22.2)	2 (16.7)	

Abbreviation: Dex, dexmedetomidine.

^a^ Values are expressed as No. (%).

#### 4.1.7. Duration of Extubation, Drug and Anesthesia Complications, and Treatment of Complications

Perioperative findings, including extubation duration, drug side effects, and anesthesia-related complications, are summarized in Table 3. The most common complication was nausea and vomiting, with no statistically significant difference between the two groups. However, the frequency of bradycardia and hypotension was significantly higher in the Dex group than in the placebo group.

**Table 3.** Comparison of Extubation Duration, Drug and Anesthesia Complications and Their Treatment in Patients of Two Study Groups ^a^

Group Variant	Dex (30 Cases)	Placebo (30 Cases)	Total	P-Value
**Extubation time (min)**	6.3 ± 1.3	6.7 ± 1.3	6.5 ± 1.3	0.215
**Drug and anesthetic side effects**				
Hypoxia	0	0	0	-
Bradycardia	8 (26.7)	0	8 (13.3)	0.002
Tachycardia	0	0	0	-
Hypotension	6 (20)	0	6 (10)	0.012
Hypertension	0	0	0	-
Nausea and vomiting	6 (20)	5 (16.7)	11 (18.3)	0.739
Drowsiness	1 (3.3)	0	1 (1.7)	0.313
Anxiety	0	1 (3.3)	1 (1.7)	0.313
**Treatment of complications**				
Pethidine	0	2 (6.7)	2 (3.3)	0.112
Atropine	2 (6.7)	0	2 (3.3)	0.112

Abbreviation: Dex, dexmedetomidine.

^a^ Values are expressed as No. (%) or mean ± SD.

## 5. Discussion

Shivering is still one of the important issues after surgery and anesthesia. The exact mechanism of shivering is not yet fully understood. Several hypotheses have been proposed in this regard, including hypothermia before surgery, postoperative pain, loss of body heat after surgery, and the direct effect of anesthetic drugs (21). Patients who undergo spine surgery in the prone position are prone to hypothermia due to the long duration of the surgery and the large exposed skin surface (22).

In the current study, which was conducted as a double-blind randomized clinical trial on 60 patients undergoing spinal surgery in the prone position, the effect of Dex in the form of a bolus dose of 1 mcg/kg in 10 ml of normal saline followed by an infusion of 0.5 mcg/kg/h was evaluated in comparison with placebo, regarding the frequency and intensity of shivering in the studied patients. The analysis of the demographic variables of the patients showed that the two study groups were similar in terms of demographic characteristics.

In the current study, the evaluation of the hemodynamic findings of patients from the start of anesthesia until after extubation showed that MAP values were significantly lower at the end of surgery, before, and after extubation in the Dex group compared to the placebo group. Regarding HR changes, it was also observed that from the 60th minute of surgery to after extubation, HR values in the Dex group were significantly lower than in the placebo group. No significant difference was observed between the two study groups in terms of SPO_2_ changes; however, RR values after extubation were significantly higher in the Dex group. Consistent with the current study, it was also observed in the study of Usta et al. that MAP and HR values were lower in patients receiving Dex compared to the placebo group (23).

Dexmedetomidine is a central alpha-2 adrenoceptor agonist with predictable analgesic and sympatholytic effects. The hemodynamic response following Dex infusion depends on the dose and rate of infusion. A transient hypertensive phase with reflex bradycardia followed by hypotension is observed at higher doses and infusion rates. The reduction in HR and BP is due to the reduction of central sympathetic outflow (24). In the study by Hall et al., which was conducted regarding the sedative, amnesic, and analgesic properties of low doses of Dex infusion, it was also mentioned that the initial bolus dose of Dex led to a transient increase in BP and reflex bradycardia, which is related to alpha-2 agonism and is mitigated by a slower infusion rate (25).

In the current study, the analysis of the changes in body temperature showed that from the beginning of the study to the end of extubation, and also in the PACU at the evaluated times, the body temperature of the patients in the Dex group was significantly higher than in the placebo group. Moreover, the increase in body temperature in the PACU over time was significantly greater in the Dex group compared to the placebo group. Meanwhile, the frequency and intensity of shivering in the Dex group were significantly lower than those in the placebo group.

In the study by Bajwa et al., who evaluated the effect of shivering in laparoscopic surgery candidates, the frequency of shivering in the Dex group was significantly lower than in the placebo group (5% versus 42.5%), consistent with the current study (26). In another study conducted by Megalla et al., comparing Dex and nalbuphine for the treatment of shivering after vaginal hysterectomy, the frequency of shivering was reported as 0% in the Dex group, 8% in the nalbuphine group, and 68% in the placebo group (27). Another study comparing Dex and ondansetron for the treatment of shivering after spinal block also found, consistent with the current study, that the frequency of shivering was significantly lower in the Dex group than in the placebo group (5.5% versus 57%) (27, 28). In the study by Bozgeyik et al., conducted on patients undergoing arthroscopy, a dose of 0.5 µg/kg of Dex was used, and shivering occurred in only 1 out of 30 patients, consistent with the current findings (28). Similarly, in the study by Karaman et al., which evaluated Dex infusion to prevent shivering in laparoscopic gynecology surgeries, a dose of 1 µg/kg was used, and the incidence of shivering was significantly lower in the Dex group compared to the placebo group (10% versus 46.6%) (29).

According to available evidence, central adrenergic receptors play an important role in the treatment of shivering caused by general anesthesia (1). Various studies have reported that clonidine, an alpha-2 receptor agonist, has acceptable efficacy in the treatment and prevention of shivering (30). Dexmedetomidine, which is similar to clonidine, is a highly selective alpha-2 receptor agonist and appears to exert its anti-shivering effect by reducing vasoconstriction and lowering the shivering threshold (31). Additionally, Dex has thermoregulatory effects on the hypothalamus. The main advantage of using Dex is its short half-life, approximately 2 - 3 hours, which allows it to be administered as a single dose or multiple doses during surgery to control shivering (29, 31).

In the current study, the most common complication was nausea and vomiting; however, no statistically significant difference was observed between the two groups. In contrast, the frequency of bradycardia and hypotension was significantly higher in the Dex group than in the placebo group. Based on these results and the alignment with previous findings, the anti-shivering effect of Dex compared to placebo is significant. Dex appears to establish this effect through its central alpha-2 agonist action, which may lead to side effects such as hypotension, bradycardia, and excessive sedation (32). In the current study, the frequency of bradycardia and hypotension was higher in patients who received Dex. In line with our findings, Prabhakaran et al. reported that while evaluating the prophylactic effect of Dex for preventing shivering under spinal anesthesia, the frequency of bradycardia and hypotension was higher in the Dex group compared to the placebo group, although the difference was not statistically significant (33).

## Data Availability

The dataset presented in the study is available on request from the corresponding author during submission or after publication.

## Clinical Trial Registration Code:

IRCT2365113914
